# Glycemic control and associated factors among type 2 diabetes mellitus patients: a cross-sectional study of Azar cohort population

**DOI:** 10.1186/s12902-023-01515-y

**Published:** 2023-12-12

**Authors:** Masoud Faghieh Dinavari, Sarvin Sanaie, Kimia Rasouli, Elnaz Faramarzi, Roghayeh Molani-Gol

**Affiliations:** 1 Liver and Gastrointestinal Diseases Research Center, Tabriz University of Medical Sciences, Tabriz, Iran; 2 Research Center for Integrative Medicine in Aging, Aging Research Institute, Tabriz University of Medical Sciences, Tabriz, Iran; 3 Student Research Committee, Tabriz University of Medical Sciences, Tabriz, Iran; 4 Nutrition Research Center, Department of Community Nutrition, Faculty of Nutrition and Food Science, Tabriz University of Medical Sciences, Tabriz, Iran

**Keywords:** Glycemic control, T2DM, Diabetes, Blood sugar, Azar cohort

## Abstract

**Background:**

Despite the growing prevalence of diabetes and its complications, there is a dearth of data regarding factors associated with glycemic control. Therefore, in this cross-sectional study, we aimed to identify factors influencing glycemic control in patients with type 2 diabetes mellitus (T2DM) in the Iranian population.

**Methods:**

This cross-sectional study was conducted among the Azar cohort population and the glycemic control status of patients with T2DM was investigated. Possible risk factors including age, sex, marital status, educational level, smoking status, sleep duration, family history of diabetes and hypertension, socioeconomic status, physical activity level, and co-existence of other chronic diseases and their relationship with glycemic control status were also assessed. Multivariate logistic regression analysis was used to identify determinants of glycemic control.

**Results:**

Among 1,710 T2DM patients (60.2% female), the overall prevalence of poor glycemic control was 56.8%. In the unadjusted logistic regression analysis model, a low wealth score index significantly increased the risk of poor glycemic control (OR: 1.49;1.10–2.02). Variables significantly associated with poor glycemic control even after adjusting for confounding factors were first-degree family history of diabetes (OR: 1.34; 1.08–1.65), and sleep duration (OR: 1.29 ;1.02–1.62 for 6.6-8 h/d; OR:1.42;1.10–1.88 for > 8 h/d). Interestingly, we found that the co-existence of ≥ 3 chronic diseases with diabetes decreased the risk of poor glycemic control.

**Conclusions:**

In the current study, most of the patients with T2DM had uncontrolled glycemic control. Due to the individual and social costs of diabetes complications, it is necessary to suggest tailored and effective interventions for controlling blood glucose levels in people with diabetes.

## Introduction

Diabetes mellitus (DM) is a global public health problem that leads to about 5 million deaths annually because of its complications [1]. In fact, type 2 diabetes mellitus (T2DM) is a lifestyle-related disease that is on the rise due to obesity, inactivity, and Westernized food intake [1]. It is estimated that more than 422 million adults are living with diabetes worldwide, projected to reach about 642 million by 2040 [1]. The burden of diabetes mostly affects resource-limited countries where screening and access to treatment and care are not readily available [2].

Poor and inadequate glycemic control constitutes a major public health problem and is a significant risk factor for the development and progression of diabetes-related complications, which can markedly increase healthcare costs of the disease and reduce life expectancy and quality of life [3, 4]. Glycemic control is considered the most effective means of preventing disease complications [5]. However, a small proportion of patients maintain their blood sugar levels below 7% glycated hemoglobin (HbA1C), the ideal target for type 2 diabetes patients; 53–70% of diabetic patients have uncontrolled blood glucose levels and require help to prevent complications and reduce mortality [6].

Some recent studies reported that good glycemic control might be attributed to availability and access to primary care, better knowledge level, presence of functional health insurance, and uniformity in assessing glycemic control [7, 8]. Moreover, other studies around the world demonstrated that glycemic control is associated with various factors such as age, gender, duration of diabetes, type of treatment, body mass index (BMI), fasting plasma glucose (FPG), lipid profile, level of education, occupation, medication adherence, presence of comorbidities, self-care practice, and mental and psychosocial health problems [9–14]. A Yemeni study showed that younger age, lack of regular exercise, lower education level, a diabetes diagnosis of more than seven years, albuminuria, and insulin treatment cause uncontrolled blood glucose levels [15]. Moreover, Fiseha et al. reported that rural residency, diabetes duration of over ten years, a low education level, and working in sales were factors of poor glycemic control amongst Ethiopian diabetes patients [16]. Recent studies have pinpointed inadequate medication adherence and a lack of compliance with scheduled follow-up appointments as factors independently linked to suboptimal glycemic control [17, 18]. Consequently, variations exist between countries and ethnic groups in terms of the contributors to uncontrolled blood glucose, and the findings regarding factors associated with poor glycemic control have been inconsistent [19]. The risk factors assessed for poor glycemic control also exhibited diversity across the various studies. Moreover, studies on the association of multimorbidity and blood glucose control are very limited; while, the majority of studies examining the correlation between multimorbidity and glycemic control have primarily concentrated on hypoglycemia [20, 21]. However, prior research suggests that uncontrolled diabetes, leading to hyperglycemia, remains the primary concern in developing countries [22, 23]. Thus, despite the growing prevalence of diabetes and its complications, there is a dearth of data regarding factors associated with glycemic control [16]. To our knowledge, the current study is the first to comprehensively assess the relationship between multimorbidity and other possibly relevant factors with blood glucose control in the Iranian population. Such data could be used for planning healthcare programs to improve diabetes control.

In this cross-sectional study, our objective was to uncover the factors that influence glycemic control in individuals with type 2 diabetes mellitus (T2DM) within the Iranian population. We posited that variables such as socioeconomic status (SES), family history of diabetes and hypertension, the duration of the disease, sleep duration, and coexisting medical conditions could potentially have a substantial impact on determining glycemic control.

## Methods

### Study design and participants

This cross-sectional study was conducted based on the Azar Cohort Study, which is a part of the Prospective Epidemiological Research Studies in Iran (PERSIAN) cohort [24]. The Azar Cohort study is explained in detail in the published cohort profile article [25]. The protocol of this study was registered the Research Vice Chancellor of Tabriz University of Medical Sciences, Tabriz, Iran and was approved by the Ethics Committee of Tabriz University of Medical Sciences (IR.TBZMED.REC.1400.621).

The AZAR cohort, which commenced in 2014, has been an ongoing endeavor since its inception. This cohort was established through three distinct phases: a pilot phase, the enrollment phase, and the continuous follow-up of participants over a span of 15 years. For the purposes of this study, data collected during the pilot and enrollment phases from 2014 to 2017 were utilized.

The inclusion and exclusion criteria for the Azar cohort study entail having a permanent residence in the Shabestar region for a minimum of nine months, providing written informed consent, and having at least one parent of Azeri parents. Individuals with mental or physical disabilities were not included in the study.

All participants were informed of the study procedure and signed a written informed consent and for all illiterate participants informed consent was obtained from their legal guardian.

The inclusion criteria for the current study encompassed individuals with type 2 diabetes who were prescribed either glucose-lowering drugs or a combination of glucose-lowering drugs along with insulin therapy. According to our specific criteria, out of the 15,006 participants in the Azar cohort study, a total of 1,710 patients with type 2 diabetes were identified and selected for this study.

### Data collection

The electronic questionnaire, which comprises a total of 55 questions and 482 items, is administered by an interviewer. This questionnaire delves into various aspects of the participant’s life that may have an impact on their health status. The questions are grouped into several key categories, including demographics, SES, lifestyle factors (such as physical activity and substance use), occupational history (including exposure to workplace hazards), past medical history, medication use (past and present), family medical history, gynecological and obstetric history (for female participants), oral and dental health, circadian rhythms, dietary habits (including food processing and cooking methods), psychiatric assessments (covering mood symptoms, personality traits, and sleep patterns), and environmental factors (such as mobile phone usage, pesticide exposure, housing conditions, and water source).

To assess dietary patterns, a validated food frequency questionnaire (FFQ) [26] is employed. This FFQ includes 130 food items, encompassing various food groups such as grains and cereals, meat and dairy products, oils, sweets, legumes, vegetables, fruits, and condiments, in addition to information about cooking methods. During the physical examination, a trained general practitioner adheres to cohort-specific guidelines.

### Wealth score index, metabolic equivalents of task, smoking status

SES was evaluated using Wealth Score Index (WSI), calculated by Multiple Correspondence Analysis (MCA). Participants of the study were categorized into five WSI quintiles, from the lowest (1st quintile) to the highest (5th quintile). A valid Self-Report Instrument was used as a questionnaire in this study [27, 28] to assess the participants’ daily activity in terms of metabolic equivalents of Task (METs). Each MET is equal to the amount of energy that a person consumes relative to their weight.

Smoking status was classified into three distinct groups. Participants were categorized as (i) “non-smokers” if they had either never smoked or had smoked fewer than 100 cigarettes in their lifetime, (ii) “ex-smokers” if they had smoked more than 100 cigarettes in their lifetime or had consumed one or more cigarettes daily but had refrained from smoking for at least one year, or (iii) “smokers” if they were currently smoking one or more cigarettes per day or using alternative tobacco products like pipes or hookahs at least once a week.

### Glycemic control definition

Based on the guidelines of the American Diabetes Association (ADA), good glycemic control in patients with diabetes was denoted by FBS values of 80–130 mg/dl [29], while poor glycemic control was considered by FBS ≥ 130 mg/dl.

### Multimorbidity definition

In addition to diabetes, the co-existence of other chronic diseases, such as hypertension, cancers, stroke, fatty liver, and cardiovascular diseases, were evaluated. Based on this information, multimorbidity (MM) was defined as the co-existence of two or more chronic diseases. A positive history of such diseases was noted if the participant recalled being told by a physician that they had the disease. A BMI of above 30 kg/m^2^ signaled obesity.

### Statistical analysis

Descriptive statistics, including frequency, percentages, mean, and standard deviation (SD), were reported for all variables of concern. The chi-squared test was used appropriately to examine the general characteristics across the features of the determined categories. Furthermore, logistic regression was conducted to analyze the predicting factors of glycemic control (Model 1: unadjusted; Model 2: adjusted for age, gender, and SES (WSI); Model 3: adjusted for age, gender, SES, and diabetes duration. The odds ratios (ORs) and related 95% confidence intervals (CIs) were evaluated. The statistical significance level was set at *P* < 0.05. Finally, SPSS software was used to analyze the data (SPSS Inc., Chicago, IL, version 20).

## Results

The prevalence of poor glycemic control in this study sample of T2DM patients was 56.8%. The characteristics of the patients with T2DM based on glycemic control are presented in Table 1. There were no significant differences between poor and good glycemic control regarding age, anthropometric measurements, gender, education level, physical activity level, smoking status, family history of diabetes and hypertension (second-degree relatives). We found that the number of patients in the first tertile of WSI with poor glycemic control was significantly higher than good glycemic control (*P* = 0.014). Of 738 patients with good glycemic control, 473 had a first-degree family history of hypertension, significantly higher than poor glycemic control (*P* = 0.011). Interestingly, stroke, chronic obstructive pulmonary disease (COPD), and rheumatoid disease were more prevalent in good glycemic control than poor glycemic control. Moreover, the percentage of patients with three or more chronic diseases in good glycemic control was significantly higher than poor glycemic control (*P* < 0.001).

**Table 1:** General characteristics of diabetic patients stratified by glycemic controlled

Characteristics	Glycemic controlled (*n* = 738)	Glycemic Not Controlled (*n* = 972)	*P
	N (%)	N (%)	
**Gender**			0.213
Male	281 (41.3)	400(58.7)	
Female	457 (44.4)	572 (55.6)	
**Marital status**			0.810
Not married	74 (42.0)	102 (58.0)	
Married	664 (43.3)	870 (56.7)	
**Education levels**			0.878
Illiterate	201 (42.9)	267 (57.1)	
Primary school	290 (42.5)	393 (57.5)	
Diploma	202 (43.5)	262 (56.5)	
University	44 (46.8)	50 (53.2)	
**BMI classification (Kg/m2)**			0.012
<18.5 Underweight	1 (25)	3 (75)	
18.5–24.9 Normal weight	84 (41.2)	120 (58.8)	
25-29.9 Overweight	253 (38.9)	398 (61.1)	
≥30 Obese	400 (47)	451 (53)	
**Physical activity level (METs**^**a**^**)**			0.529
Low	351 (44.6)	436 (55.4)	
Moderate	225 (41.7)	315 (58.3)	
High	162 (42.3)	221 (57.7)	
**Quintiles of wealth index**			0.014
1(poorest)	162 (36.7)	279 (63.3)	
2	162 (44.6)	201 (55.4)	
3	159 (42.9)	212 (57.1)	
4	125 (49)	130 (51)	
5 (richest)	130 (46.4)	150 (53.6)	
**Current smoking status**			0.833
Non smoker	575 (43.5)	746 (56.5)	
Ex-smoker	74 (41.3)	105 (58.7)	
Smoker	89 (42.4)	121 (57.6)	
**Family history of diabetes (first degree relative)**	453 (40.2)	673 (59.8)	0.001
**Family history of diabetes (second-degree relative)**	210 (40.1)	314 (59.9)	0.090
**Family history of hypertension (first degree relative)**	473 (45.7)	563 (54.3)	0.011
**Family history of hypertension (second degree relative)**	122 (40.0)	183 (60.0)	0.226
**Diabetes duration (years)**			< 0.001
<5	480 (51.7)	448 (48.3)	
5–10	164 (31.5)	357 (68.5)	
>10	94 (36)	167 (64)	
**Sleep duration (hours/day)**			0.015
≤6.5	275 (48.0)	298 (52.0)	
6.6-8	289 (41.3)	411 (58.7)	
>8	174 (39.8)	263(60.2)	
**Common chronic diseases**			
Hypertension	366 (45.6)	437 (54.4)	0.063
Cancers	5 (45.5)	6 (54.5)	0.87
Fatty liver	91 (48.9)	95 (51.1)	0.093
Stroke	15 (65.2)	8 (34.8)	0.032
CVD	92 (43)	122 (57)	0.95
COPD	51 (60)	34 (40)	0.002
Rheumatoid	42 (56)	33 (44)	0.024
Obesity	400 (47)	451 (53)	0.001
**Number of chronic Diseases**			< 0.001
1	130 (35.3)	238 (64.7)	
2	215 (40.8)	312 (59.2)	
3	221 (46.3)	256 (53.7)	
≥4	172 (50.9)	166 (49.1)	
	**Mean ± SD**	**Mean ± SD**	**P
**Age (years)**	55.50 ± 8.22	55.28 ± 7.97	0.588
**Height (cm)**	160.22 ± 9.53	160.69 ± 9.15	0.302
**Weight (kg)**	78.57 ± 14.31	77.76 ± 14.10	0.242
**Waist circumference**	99.70 ± 10.64	99.96 ± 10.48	0.614

*P: chi –square, **P :independent-t-test

^a^METs: metabolic equivalents of Task

As indicated in Table 2, in the unadjusted model, a low WSI significantly increased the risk of poor glycemic control (OR:1.49;1.10–2.02). In addition, a first-degree family history of diabetes increased the risk of poor glycemic control, while a first-degree family history of hypertension had a protective effect against poor glycemic control. These associations remained significant even after adjusting for confounding factors. Our results indicated that diabetic patients were more likely to have poorly controlled diabetes if they had a longer diabetes duration (adjusted model2 OR: 2.50; 1.99–3.15 for 5–10 years and OR: 2.09; 1.56–2.81 for > 10 years), sleep more than 6.5 h/d (adjusted model 3 OR:1.29; 1.02–1.62 for 6.5-8 h/d and OR: 1.42; 1.10–1.85 for > 8 h/d). Moreover, diabetic patients with a history of hypertension, stroke and COPD had a low risk of poor glycemic control. Simultaneously, the co-existence of ≥ 3 chronic diseases with diabetes decreased the risk of poor glycemic control.

**Table 2:** Predictor factors for glycemic control in the Azar cohort population

Characteristics	Unadjusted OR (95%CI)	P*	Adjusted OR (95%CI)^a^	P*	Adjusted OR (95%CI)^b^	P*
**Age (years)**	0.99(0.98-1.00)	0.587	-		-	
**Gender**						
Male	1.13 (0.93–1.38)	0.20	-	-	-	
Female	Reference		-	-	-	
**Quintiles of wealth index**						
1(poorest)	1.49(1.10–2.02)	0.010	-	-	-	
2	1.07(0.78–1.47)	0.649	-	-		
3	1.15(0.84–1.57)	0.364	-	-	-	
4	0.90(0.64–1.26)	0.549	-	-	-	
5 (richest)	reference					
**Education level**						
Illiterate	1.16(0.74–1.82)	0.491	1.16(0.70–1.92)	0.560	1.20(0.71–2.01)	0.489
Primary school	1.19(0.77–1.83)	0.425	1.16(0.73–1.85)	0.518	1.19(0.74–1.93)	0.460
Diploma	1.14(0.73–1.78)	0.560	1.14(0.72–1.81)	0568	1.15(0.72–1.84)	0.553
University	reference					
**BMI classification (Kg/m2)**						
18.5–24.9 normal weight	reference					
25-29.9 Overweight	1.10(0.79–1.51)	0.555	1.09(0.79–1.51)	0.577	1.10(0.79–1.54)	0.543
≥30 obese	0.78 (0.58–1.07)	0.134	0.78(0.57–1.08)	0.140	0.82(0.59–1.14)	0.247
**Physical activity level (METs**^**a**^**)**						
Low	0.91(0.71–1.16)	0.457	0.97(0.75–1.26)	0.848	0.98(0.75–1.27)	0.878
Moderate	1.02(0.75–1.38)	0.897	1.11(0.84–1.46)	0.451	1.16(0.87–1.53)	0.304
High	Reference					
**Current smoking status**						
Non smoker	Reference					
Ex-smoker	1.09(0.79–1.50)	0.580	1.00(0.69–1.45)	0.971	1.02(0.70–1.49)	0.883
Smoker	1.04(0.78–1.40)	0.755	0.92(0.65–1.31)	0.665	0.96(0.67–1.37)	0.854
**Family history of diabetes (first-degree relative)**	1.41(1.15–1.73)	0.001	1.47(1.19–1.80)	< 0.001	1.34(1.08–1.65)	0.006
**Family history of diabetes (second-degree relative)**	1.20(0.97–1.47)	0.087	1.25(1.00-1.55)	0.043	1.14(0.91–1.43)	0.224
**Family history of hypertension (first-degree relative)**	0.77(0.63–0.93)	0.010	0.80(0.66–0.98)	0.038	0.78(0.63–0.95)	0.018
**Family history of hypertension (second-degree relative)**	1.17(0.91–1.50)	0.220	1.25(0.96–1.62)	0.086	1.15(0.88–1.50)	0.286
**Diabetes duration (years)**						
<5						
5–10	2.33(1.86–2.92)	< 0.001	2.50(1.99–3.15)	< 0.001	-	
>10	1.90(1.43–2.52)	< 0.001	2.09(1.56–2.81)	< 0.001	-	
**Sleep duration (hours/day)**						
≤6.5	Reference					
6.6-8	1.31(1.05–1.63)	0.017	1.31(1.05–1.65)	0.015	1.29(1.02–1.62)	0.028
>8	1.39(1.08–1.79)	0.010	1.40(1.09–1.82)	0.009	1.42(1.10–1.85)	0.008
**Common chronic diseases**						
Hypertension	0.83(0.68-1.00)	0.057	0.84(0.68–1.03)	0.099	0.80(0.65–0.99)	0.047
Cancers	0.91(0.27–2.99)	0.877	0.87(0.26–2.91)	0.834	0.84(0.24–2.85)	0.779
Fatty liver	0.77(0.56–1.04)	0.093	0.79(0.58–1.07)	0.135	0.78(0.57–1.08)	0.139
Stroke	0.40(0.16–0.94)	0.038	0.40(0.16–0.96)	0.042	0.36(0.14–0.87)	0.025
CVD	1.00(0.75–1.34)	0.958	0.99(0.73–1.33)	0.950	1.00(0.73–1.35)	1.00
COPD	0.48(0.31–0.76)	0.002	0.50(0.32–0.79)	0.003	0.52(0.33–0.83)	0.006
Rheumatoid	0.58(0.36–0.92)	0.023	0.64(0.39–1.02)	0.065	0.66(0.41–1.08)	0.099
**Number of chronic Diseases**						
1	Reference					
2	0.79(0.60–1.04)	0.098	0.81(0.61–1.07)	0.142	0.8(0.61–1.09)	0.183
3	0.63(0.47–0.83)	0.001	0.63(0.47–0.84)	0.002	0.65(0.48–0.87)	0.004
≥4	0.53(0.39–0.71)	< 0.001	0.54(0.39–0.74)	< 0.001	0.53(0.38–0.74)	< 0.001

*OR* Odd Ratio, *CI* Confidence Interval

*P < 0.05 is significant

^a^Adjusted for age, gender, and socioeconomic status

^b^Adjusted for age, gender, socioeconomic status and diabetes duration

## Discussion

Diabetes is increasing in prevalence globally due to changes in aging, sedentary lifestyle, poor diet, lack of medical information and medical access, and other risk factors [30]. To prevent diabetes-related complications, several factors relevant to poor glycemic control must be recognized and managed. In the current study, we investigated the glycemic control status and its influential factors in diabetic patients in the Azar cohort population. We found that most patients (56.8%) had poor glycemic control status. Consistent with our findings, Babaniamansour et al. [31] also found that 49% of the 562 subjects diagnosed with T2DM exhibited inadequate glycemic control. Recently, based on STEPS survey in Iran, poor glycemic control has been reported in 51.4% of diabetic patients [23] that was roughly the same as our study. Alzaheb et al. conducted a study on adult patients with T2DM in Saudi Arabia and reported a higher prevalence of poor glycemic control compared to the results of our study [32]. They found that a substantial 74.9% of the patients in their study had inadequate blood glycemic control [32].

Nevertheless, other studies have presented different outcomes. For instance, in the United States, the rate of uncontrolled blood glucose was reported at 35% [33], and in Europe, it was found to be 37% [34]. When comparing these figures with the high prevalence of poor glycemic control in Iran and other developing countries [35], it underscores the urgency of intensifying efforts to enhance glycemic control among individuals with T2DM.

Recent studies have suggested that the diversity in glycemic control status can be attributed to variations in sample size, the extent of medication adherence, the duration of the disease [36–38], as well as differences in disease awareness and suboptimal treatment in developing countries [39–41].

The results of this study also demonstrated a significant difference between T2DM patients with good and poor glycemic control in factors such as wealth index, family history of diabetes and hypertension, diabetes and sleep duration, and some chronic diseases. As such, the prevalence of poorest socioeconomic level, first-degree family history of diabetes, diabetes duration of 5–10 years, and sleep duration of more than 6.5 h/d were higher in people with uncontrolled glycemic levels. The logistic regression analysis results also confirmed these findings, indicating that some variables had a significant role in poor glycemic control, including poor SES, family history of diabetes, diabetes duration, and sleep duration.

Based on the findings, individuals with poor SES had a significantly increased risk of poor glycemic control. In line with our findings, Rahman et al. [42] and Babaniamansour et al. [31] have noted that glycemic control tends to be more favorable in patients with T2DM of lower SES compared to those with higher SES. However, a recent study by Yahaya et al. found no significant association between patients’ SES and poor glycemic control [18]. It’s worth noting that several other studies have also reported a similar link between socioeconomic level and glycemic control [42–44].

The relationship between low SES and poor glycemic control is probably because of mediating variables such as comorbid conditions, adverse health-related behaviors, and non-adherence to essential health service-related practices. Social factors play a decisive role in maintaining health-related behavior [45]. Jaffiol et al. [46] demonstrated that low SES was associated with undesirable diabetes meal patterns. According to their financial affordability, people within the low SES cluster consumed a large amount of carbohydrates and fewer super molecules, vegetables, and contemporary fruits. These results may provide insight into the development of means to improve glycemic control and reduce the incidence of chronic complications among patients with low SES.

The results of this study indicated that the patients with a family history of diabetes in first-degree relatives had a significantly higher risk of poor glycemic control compared to the good glycemic control. Consistent with our research, Haghighatpanah et al. [47] and Alzaheb et al. [32] have likewise found that patients with T2DM who have a family history of diabetes tend to exhibit poorer glycemic control. A possible explanation of why patients with a history of diabetes are at greater risk of having poor glycemic control is that the disease has inherent genetic risk factors that have the power to influence its severity and duration [48].

The current study also uncovered that having a sleep duration exceeding 6.5 h per day was linked to poor glycemic control. In a similar vein, Bawadi et al. [49] conducted a study involving 2,500 participants aged 18 to 60 years and found that sleeping for an extended duration at night, specifically 8 h or more, increased the risk of experiencing poor glycemic control. Sleep duration is suggested to be related to glycemic control in people with diabetes. The bidirectional relationship between sleep disorders and metabolic disease has been shown by several studies [50–53]. Previous studies reported that short and long sleep durations were related to high HbA1c levels in patients with diabetes [54, 55] with a U-shaped dose-response relationship [56]. Some studies suggest that this correlation is mediated by the impact of sleep duration on appetite-regulating hormones [57]. However, the mechanism behind the link between long sleep duration and hyperglycemia remains elusive and needs further investigation [55].

The findings of the present study suggest that a diabetes duration of more than five years is linked to poor glycemic control. This aligns with several other studies that have shown a significant association between glycemic control and the duration of diabetes [19, 35, 46, 58–60]. For example, Ahn et al. [19] conducted a study involving 522 rural diabetic subjects and demonstrated that individuals with a diabetes duration exceeding seven years had notably higher rates of poor glycemic control compared to their counterparts. Similarly, studies by Khattab et al. [5] and Eid et al. [61] have indicated that a longer duration of T2DM is strongly correlated with suboptimal glycemic control. However, it’s worth noting that in the study by Yahaya et al., the duration of diabetes treatment was not found to be associated with poor glycemic control among the patients in their sample [18]. These findings are perhaps due to excessive insulin resistance or the progressive restriction of insulin secretion over time caused by B-cell failure, which means that a patient’s positive response to oral pharmacological agents or dietary changes is less likely [62, 63].

Regarding the prevalence of chronic diseases and the number of chronic diseases in patients with poor glycemic control, we obtained interesting findings. In the current study, the prevalence of stroke, COPD, rheumatoid disease, and obesity was higher in subjects with good glycemic control. Moreover, the percentage of patients with three or more chronic diseases was significantly higher in good glycemic control than in poor glycemic control (*P* < 0.001). In line with our own findings, McCoy et al. conducted a study involving 194,157 patients diagnosed with T2DM and observed that the greatest proportion of patients who achieved low HbA1c levels were those with multiple coexisting medical conditions (referred to as multimorbid patients) [64]. However, some previous studies reported inconsistent results; for example, Haghighatpanah et al. [47] indicated that the presence of comorbidity was similar in individuals with good and poor glycemic control. Prior studies were varied in considering the presence of comorbidity and specific comorbidities as a reflection of heightened poor glycemic control risk. The possible explanation for the association between multimorbidity and improved glycemic control may be attributed to more effective healthcare management for patients dealing with multiple chronic conditions. In other words, the presence of comorbidities might enhance the opportunities for comprehensive treatment and the desired level of diabetes control. It’s worth noting that uncontrolled diabetes can also increase the risk of developing these concurrent conditions.

Additionally, patients with multimorbidity are often more likely to be treated with insulin in order to achieve lower HbA1c levels. In contrast, individuals who are generally healthier but have elevated HbA1c levels are less frequently prescribed insulin, even if they have suboptimal glycemic control. This could be because the presence of multiple comorbidities prompts a shared approach to treatment goals and fosters the potential for collaborative disease management. As a result, more intensive strategies for lowering glucose levels, including insulin therapy, may be considered in cases of cumulative multimorbidity [65].

With all these, recognizing the comorbidity burden at the population level may be an effective and simple way of identifying the highest-risk individuals in need of closer monitoring and potential interventions [66].

The achievements of this research have some implications. These findings highlight the need for appropriate management of glycemic control in T2DM patients. On the other hand, making positive lifestyle changes in individuals with T2DM can significantly contribute to better glycemic control and help prevent the onset of related complications. Moreover, by identifying the associated factors with poor glycemic control, healthcare providers can better focus on these identified risk factors in their health strategies, so that they can better help to improve glycemic control among diabetes patients. Health professionals and policymakers can also utilize these findings to promote health and raise awareness about factors associated with poor glycemic control through educational interventions and the formulation of suitable policies.

However, it is important to acknowledge that this study has some limitations. For instance, it employed a cross-sectional design, which makes it challenging to establish a clear cause-and-effect relationship between glycemic control and the various potential factors that influence it. Additionally, most of the data relied on self-reporting, which introduces the possibility of recall bias due to its subjective nature. Furthermore, the study used FBS as the criterion for defining glycemic control and did not take into account HbA1C levels, which may have led to an underestimation of the prevalence of poor glycemic control. Nonetheless, the current study had a large sample size and evaluated numerous relevant factors and diseases, making a meaningful contribution to comprehensively identifying the factors affecting blood glucose control.

## Conclusions

In the present study, most of the patients with T2DM had uncontrolled glycemic control. In relation to the factors affecting glycemic control in this population, our study identified several predictors of poor glycemic control, including lower SES, a first-degree family history of diabetes, a diabetes duration of over five years, and a sleep duration exceeding 6.5 h per day. Notably, patients with multiple concurrent medical conditions (multimorbidity) exhibited better glycemic control, possibly due to the comprehensive healthcare they receive for their comorbidities.

Considering the substantial individual and societal costs associated with diabetes complications and the critical role of glycemic status in the development of these complications, it is crucial to identify the relevant factors. The findings of this study can serve as a valuable resource for shaping interventions and strategies aimed at enhancing glycemic control and preventing complications in individuals with T2DM. Tailored and effective interventions for managing blood glucose levels in diabetic patients are essential. Further research in this field, especially in diverse populations, is needed to validate and expand upon these findings and to explore additional potentially influential factors.

## Data Availability

The data that support the findings of this study are available from Vice Chancellor for Research but restrictions apply to the availability of these data, which were used under license for the current study, and so are not publicly available. Data are however available from the authors upon reasonable request and with permission of Vice Chancellor for Research.

## References

[1] Atlas D. International diabetes federation. IDF Diabetes Atlas, 7th edn. Brussels, Belgium: International Diabetes Federation. 2015;33(2).

[2] Shaw JE, Sicree RA, Zimmet PZ (2010). Global estimates of the prevalence of diabetes for 2010 and 2030. Diabetes Res Clin Pract.

[3] Lloyd A, Sawyer W, Hopkinson P (2001). Impact of long-term complications on quality of life in patients with type 2 diabetes not using insulin. Value Health.

[4] Stratton IM, Adler AI, Neil HA, Matthews DR, Manley SE, Cull CA, et al. Association of glycaemia with macrovascular and microvascular complications of type 2 diabetes (UKPDS 35): prospective observational study. Bmj. 2000;321(7258):405–12. 10.1136/bmj.321.7258.405 PMC27454 10938048

[5] Khattab M, Khader YS, Al-Khawaldeh A, Ajlouni KJJD (2010). Complications i. Factors associated with poor glycemic control among patients with type 2. Diabetes.

[6] Buchanan D (2003). Social epidemiology: Berkman, LF, Kawachi I (Eds) Oxford University Press, New York, 2000, pp. 391.

[7] Ali MK, McKeever Bullard K, Imperatore G, Barker L, Gregg EW (2012). Characteristics associated with poor glycemic control among adults with self-reported diagnosed diabetes—National Health and Nutrition Examination Survey, United States, 2007–2010. MMWR Morb Mortal Wkly Rep.

[8] Islam SM, Niessen LW, Seissler J, Ferrari U, Biswas T, Islam A, Lechner A. Diabetes knowledge and glycemic control among patients with type 2 diabetes in Bangladesh. SpringerPlus. 2015;4:1–7. 10.1186/s40064-015-1103-7 PMC4474969 26101736

[9] Adham M, Froelicher ES, Batieha A, Ajlouni K (2010). Glycaemic control and its associated factors in type 2 diabetic patients in Amman, Jordan. East Mediterr Health J.

[10] Dhillon H, Nordin RB, Ramadas A (2019). Quality of life and associated factors among primary care Asian patients with type 2 diabetes mellitus. Int J Environ Res Public Health.

[11] Ufuoma C, Godwin YD, Kester AD, Ngozi JC (2016). Determinants of glycemic control among persons with type 2 diabetes mellitus in Niger Delta. Sahel Med J.

[12] Olayemi IA, Osazuwa F (2014). Prevalence of poor glycemic control among Nigerian female diabetics. Nig J Exp Clin Biosci.

[13] Al Mansari A, Obeid Y, Islam N, Fariduddin M, Hassoun A, Djaballah K, et al. GOAL study: clinical and non-clinical predictive factors for achieving glycemic control in people with type 2 diabetes in real clinical practice. BMJ Open Diabetes Research and Care. 2018;6(1):e000519. 10.1136/bmjdrc-2018-000519 PMC6045741 30023075

[14] Reidpath DD, Soyiri I, Jahan NK, Mohan D, Ahmad B, Ahmad MP, Kassim ZB, Allotey P. Poor glycaemic control and its metabolic and demographic risk factors in a Malaysian community-based study. Int J Public Healt. 2018;63:193–202. 10.1007/s00038-017-1072-4 29372287

[15] Saghir SA, Alhariri AE, Alkubat SA, Almiamn AA, Aladaileh SH, Alyousefi NA (2019). Factors associated with poor glycemic control among type-2 diabetes mellitus patients in Yemen. Trop J Pharm Res.

[16] Fiseha T, Alemayehu E, Kassahun W, Adamu A, Gebreweld A (2018). Factors associated with glycemic control among diabetic adult out-patients in Northeast Ethiopia. BMC Res Notes.

[17] Jarab S, Al-Qerem A, Hamam WA, Abu Heshmeh H, Al-Azzam S, Mukattash SL, Alefishat T (2023). Glycemic control and its associated factors among diabetic heart failure outpatients at two major hospitals in Jordan. PLoS One.

[18] Yahaya JJ, Doya IF, Morgan ED, Ngaiza AI, Bintabara D (2023). Poor glycemic control and associated factors among patients with type 2 diabetes mellitus: a cross-sectional study. Sci Rep.

[19] Ahn J, Yang Y. Factors associated with poor glycemic control amongst rural residents with diabetes in Korea. InHealthcare. 2021;9(4):391. 10.3390/healthcare9040391 PMC8065919 33915834

[20] Paradox of glycemic (2020). Management: multimorbidity, glycemic control, and high-risk medication use among adults with diabetes. BMJ Open Diab Res Care.

[21] Koto R, Nakajima A, Miwa T, Sugimoto K. Multimorbidity, polypharmacy, severe hypoglycemia, and glycemic control in patients using glucose-lowering drugs for type 2 diabetes: a retrospective cohort study using health insurance claims in Japan. Diabetes Ther. 2023;17:1–8. 10.1007/s13300-023-01421-5 PMC10241751 37195511

[22] Aschner P, Galstyan G, Yavuz DG (2021). Glycemic control and prevention of diabetic complications in low- and middle-income countries: an expert opinion. Diabetes Ther.

[23] Azadnajafabad S, Ahmadi N, Rezaei N (2023). Evaluation of the diabetes care cascade and compliance with WHO global coverage targets in Iran based on STEPS survey 2021. Sci Rep.

[24] Poustchi H, Eghtesad S, Kamangar F, Etemadi A, Keshtkar A-A, Hekmatdoost A (2018). Prospective epidemiological research studies in Iran (the PERSIAN Cohort Study): rationale, objectives, and design. Am J Epidemiol.

[25] Farhang S, Faramarzi E, Amini Sani N, Poustchi H, Ostadrahimi A, Alizadeh BZ (2019). Cohort profile: the AZAR cohort, a health-oriented research model in areas of major environmental change in Central Asia. Int J Epidemiol.

[26] Validity and reproducibility of a food frequency questionnaire assessing food group intake in the PERSIAN cohort study. Front Nutr. 2023;10:1059870. 10.3389/fnut.2023.1059870. eCollection 2023. 10.3389/fnut.2023.1059870 PMC10436288 37599697

[27] Validation of a New Self-Report Instrument for Measuring Physical Activity (2003). Med Sci Sports Exerc.

[28] Compendium of physical activities: an update of activity codes and MET intensities. Med Sci Sports Exerc. 2000;32(9 Suppl):S498-504. 10.1097/00005768-200009001-00009. 10.1097/00005768-200009001-00009 10993420

[29] American Diabetes Association (2023). 6. Glycemic targets: standards of care in diabetes—2023. Diabetes Care.

[30] Aung WP, Htet AS, Bjertness E, Stigum H, Chongsuvivatwong V, Kjøllesdal MKR (2018). Urban–rural differences in the prevalence of diabetes mellitus among 25–74 year-old adults of the Yangon Region, Myanmar: two cross-sectional studies. BMJ Open.

[31] Babaniamansour S, Aliniagerdroudbari E, Niroomand M (2020). Glycemic control and associated factors among Iranian population with type 2 diabetes mellitus: a cross-sectional study. J Diabetes Metab Disord.

[32] Alzaheb RA, Altemani AH (2018). The prevalence and determinants of poor glycemic control among adults with type 2 diabetes mellitus in Saudi Arabia. Diabetes Metab Syndr Obes.

[33] Laiteerapong N, Fairchild PC, Chou C-H, Chin MH, Huang ES (2015). Revisiting disparities in quality of care among US adults with diabetes in the era of individualized care, NHANES 2007–2010. Med Care.

[34] de Pablos‐Velasco P, Parhofer KG, Bradley C, Eschwège E, Gönder-Frederick L, Maheux P, et al. Current level of glycaemic control and its associated factors in patients with type 2 diabetes across Europe: data from the PANORAMA study. Clin Endocrinol. 2014;80(1):47–56. 10.1111/cen.12119 23194193

[35] Aschner P, Gagliardino JJ, Ilkova H, Lavalle F, Ramachandran A, Mbanya JC, et al. Persistent poor glycaemic control in individuals with type 2 Diabetes in developing countries: 12 years of real-world evidence of the International Diabetes Management Practices Study (IDMPS). Diabetologia. 2020;63:711–21. 10.1007/s00125-019-05078-3. 10.1007/s00125-019-05078-3 PMC7054372 31901950

[36] Abdullah A, Alkandari A, Longenecker JC, Devarajan S, Alkhatib A, Al-Wotayan R, Al-Duwairi Q, Tuomilehto J. Glycemic control in Kuwaiti diabetes patients treated with glucose-lowering medication. Prim Care Diabetes. 2020;14(4):311–6. 10.1016/j.pcd.2019.12.001 31911041

[37] Pokhrel S, Shrestha S, Timilsina A, Sapkota M, Bhatt MP, Pardhe BD (2019). Self-care adherence and barriers to good glycaemic control in Nepalese type 2 diabetes mellitus patients: a hospital-based cross-sectional study. J Multidiscip Healthc.

[38] Palella E, Cimino R, Pullano SA, Fiorillo AS, Gulletta E, Brunetti A, Foti DP, Greco M. Laboratory parameters of hemostasis, adhesion molecules, and inflammation in type 2 diabetes mellitus: correlation with glycemic control. Int J Environ Res Public Health. 2020;17(1):300. 10.3390/ijerph17010300 PMC6982208 31906326

[39] Morris-Paxton AA, Rheeder P, Ewing RMG, Ewing D (2018). Detection, referral and control of diabetes and hypertension in the rural Eastern Cape Province of South Africa by community health outreach workers in the rural primary healthcare project: health in every hut. Afr J Prim Health Care Fam Med.

[40] Fiagbe J, Bosoka S, Opong J, Takramah W, Axame W, Owusu R, et al. Prevalence of controlled and uncontrolled diabetes mellitus and associated factors of controlled diabetes among diabetic adults in the hohoe municipality of Ghana. Diabetes Management. 2017;7(5):343–54.

[41] Awadalla H, Noor SK, Elmadhoun WM, Almobarak AO, Elmak NE, Abdelaziz SI (2017). Diabetes complications in Sudanese individuals with type 2 diabetes: overlooked problems in sub-Saharan Africa?. Diabetes Metab Syndr.

[42] Rahman M, Nakamura K, Hasan S, Seino K, Mostofa G (2020). Mediators of the association between low socioeconomic status and poor glycemic control among type 2 diabetics in Bangladesh. Sci Rep.

[43] Khoda MME, Shimu IJ, Mitra P, Hossain MJ, Sarkar MS, Saha AK (2018). Modifiable factors associated with uncontrolled type 2 diabetes mellitus: experience in a tertiary care hospital of Bangladesh. BIRDEM Med J.

[44] Tao X, Li J, Zhu X, Zhao B, Sun J, Ji L, et al. Association between socioeconomic status and metabolic control and diabetes complications: a cross-sectional nationwide study in Chinese adults with type 2 diabetes mellitus. Cardiovasc Diabetol. 2016;15:1–0. 10.1186/s12933-016-0376-7 PMC4822246 27048217

[45] Emmons KM (2000). Health behaviors in a social context. Soc Epidemiol.

[46] Jaffiol C, Fontbonne A, Vannereau D, Olive J-P, Passeron S (2012). Diabetes and social deprivation. Bull Acad Natl Med.

[47] Haghighatpanah M, Nejad ASM, Haghighatpanah M, Thunga G, Mallayasamy S (2018). Factors that correlate with poor glycemic control in type 2 diabetes mellitus patients with complications. Osong Public Health Res Perspect.

[48] Gong L, Kao WHL, Brancati FL, Batts-Turner M, Gary TL (2008). Association between parental history of type 2 diabetes and glycemic control in urban African Americans. Diabetes Care.

[49] Bawadi H, Al Sada A, Al Mansoori N, Al Mannai S, Hamdan A, Shi Z, et al. Sleeping duration, napping and snoring in association with diabetes control among patients with diabetes in Qatar. Int J Environ Res Public Health. 2021;18(8):4017. 10.3390/ijerph18084017 PMC8069870 33921201

[50] Barone MT, Menna-Barreto L (2011). Diabetes and sleep: a complex cause-and-effect relationship. Diabetes Res Clin Pract.

[51] Gabryelska A, Karuga FF, Szmyd B, Białasiewicz P (2020). HIF-1α as a mediator of insulin resistance, T2DM, and its complications: potential links with obstructive sleep apnea. Front Physiol.

[52] Nicholl DD, Ahmed SB, Loewen AH, Hemmelgarn BR, Sola DY, Beecroft JM, et al. Declining kidney function increases the prevalence of sleep apnea and nocturnal hypoxia. 2012;141(6):1422-30. 10.1378/chest.11-1809 22222188

[53] Afroz-Hossain A, Dawkins M, Myers AK (2019). Sleep and environmental factors affecting glycemic control in people with type 2 diabetes mellitus. Curr Diab Rep.

[54] Sakamoto R, Yamakawa T, Takahashi K, Suzuki J, Shinoda MM, Sakamaki K (2018). Association of usual sleep quality and glycemic control in type 2 diabetes in Japanese: a cross sectional study. Sleep and Food Registry in Kanagawa (SOREKA).

[55] Zheng Y, Wang A, Pan C, Lu J, Dou J, Lu Z, et al. Impact of night sleep duration on glycemic and triglyceride levels in C hinese with different glycemic status 不同血糖水平的中国人群的夜晚睡眠时间对血糖和甘油三酯水平的影响. 2015;7(1):24–30. 10.1111/1753-0407.12186 24981368

[56] Lee SWH, Ng KY, Chin WK (2017). The impact of sleep amount and sleep quality on glycemic control in type 2 diabetes: a systematic review and meta-analysis. Sleep Med Rev.

[57] Spiegel K, Leproult R, L’Hermite-Balériaux M, Copinschi G, Penev PD, Van Cauter E (2004). Leptin levels are dependent on sleep duration: relationships with sympathovagal balance, carbohydrate regulation, cortisol, and thyrotropin. J Clin Endocrinol Metab.

[58] Ahmad NS, Islahudin F, Paraidathathu TJ (2014). Factors associated with good glycemic control among patients with type 2 diabetes mellitus. J Diabetes Investig.

[59] Gebermariam AD, Tiruneh SA, Ayele AA, Tegegn HG, Ayele BA, Engidaw M (2020). Level of glycemic control and its associated factors among type II diabetic patients in debre tabor general hospital, northwest Ethiopia. Metabol Open.

[60] Ghazanfari Z, Niknami S, Ghofranipour F, Larijani B, Agha-Alinejad H, Montazeri A (2010). Determinants of glycemic control in female diabetic patients: a study from Iran. Lipids Health Dis.

[61] Eid M, Mafauzy M, Faridah A (2003). Glycaemic control of type 2 diabetic patients on follow up at Hospital Universiti Sains Malaysia. Malays J Med Sci.

[62] Juarez D, Sentell T, Tokumaru S, Goo R, Davis J, Mau M (2006). Factors associated with poor glycemic control or wide glycemic variability among diabetes patients in Hawaii. Prev Chronic Dis.

[63] UK Prospective Diabetes Study (UKPDS) Group. Intensive blood-glucose control with sulphonylureas or insulin compared with conventional treatment and risk of complications in patients with type 2 diabetes (UKPDS 33). The lancet. 1998;352(9131):837–53. 9742976

[64] McCoy RG, Lipska KJ, Van Houten HK, Shah ND (2020). Paradox of glycemic management: multimorbidity, glycemic control, and high-risk medication use among adults with diabetes. BMJ Open Diabetes Res Care.

[65] Akın S, Bölük C (2020). Prevalence of comorbidities in patients with type–2 diabetes mellitus. Prim Care Diabetes.

[66] McCoy RG, Lipska KJ, Van Houten HK, Shah ND (2020). Association of cumulative multimorbidity, glycemic control, and medication use with hypoglycemia-related emergency department visits and hospitalizations among adults with diabetes. JAMA Netw Open.

